# Supplementary material for: Prediction of the Malignancy of a Breast Lesion Detected on Breast Ultrasound: Radiomics Applied to Clinical Practice
Source: Cancers (Basel). 2023 Feb 2;15(3):964. doi: 10.3390/cancers15030964 (PMC9913654; doi:10.3390/cancers15030964)
Supplement: Supplementary file 1 [file cancers-15-00964-s001.zip › cancers-2106145-supplementary.pdf]

cancers MDPI

Supplementary Materials

Prediction of the Malignancy of a Breast Lesion Detected on Breast Ultrasound: Radiomics Applied to Clinical Practice

Luca Nicosia, Filippo Pesapane, Anna Carla Bozzini, Antuono Latronico, Anna Rotili, Federica Ferrari, Giulia Signorelli, Sara Raimondi, Silvano Vignati, Aurora Gaeta, Federica Bellerba, Daniela Origgi, Paolo De Marco, Giuseppe Castiglione Minischetti, Claudia Sangalli, Marta Montesano, Simone Palma and Enrico Cassano

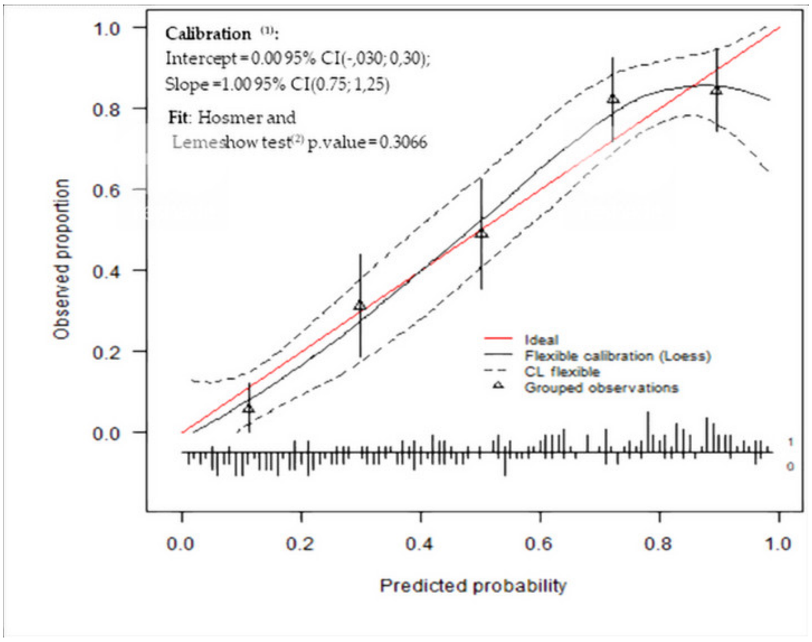

Figure S1. Evaluation of good fitting of radiomic model with Hosmer test and training group. Calibration of radiomic model. (1) Calibration intercept: a value close to 0 indicates good calibration. Calibration slope: a value close to 1 denotes perfect calibration. (2) Hosmer test: p-value > 0.05 evidence of good fit.

Table S1. Strength of agreement among four radiologists (Fleiss's K).

Operators	Estimate	Lower	Upper
Expert Operators 1 vs 2	0.8791	0.8248	0.9235
Less experienced operators 3 vs 4	0.8303	0.7726	0.885
Operator 1 vs 3	0.7145	0.6404	0.7862
Operator 2 vs 3	0.7146	0.6334	0.7911
Operator 1 vs 4	0.6664	0.5874	0.7479
Operator 2 vs 4	0.6444	0.5606	0.7259
Inter-rater Agreement (Fleiss's K)	0.7413	0.6892	0.7936
